# Mutualism Disruption Threatens Global Plant Biodiversity: A Systematic Review

**DOI:** 10.1371/journal.pone.0066993

**Published:** 2013-06-19

**Authors:** Clare E. Aslan, Erika S. Zavaleta, Bernie Tershy, Donald Croll

**Affiliations:** 1 Department of Environmental Studies, University of California Santa Cruz, Santa Cruz, California, United States of America; 2 Department of Ecology and Evolutionary Biology, University of California Santa Cruz, Santa Cruz, California, United States of America; University of Copenhagen, Denmark

## Abstract

**Background:**

As global environmental change accelerates, biodiversity losses can disrupt interspecific interactions. Extinctions of mutualist partners can create “widow” species, which may face reduced ecological fitness. Hypothetically, such mutualism disruptions could have cascading effects on biodiversity by causing additional species coextinctions. However, the scope of this problem – the magnitude of biodiversity that may lose mutualist partners and the consequences of these losses – remains unknown.

**Methodology/Principal Findings:**

We conducted a systematic review and synthesis of data from a broad range of sources to estimate the threat posed by vertebrate extinctions to the global biodiversity of vertebrate-dispersed and -pollinated plants. Though enormous research gaps persist, our analysis identified Africa, Asia, the Caribbean, and global oceanic islands as geographic regions at particular risk of disruption of these mutualisms; within these regions, percentages of plant species likely affected range from 2.1–4.5%. Widowed plants are likely to experience reproductive declines of 40–58%, potentially threatening their persistence in the context of other global change stresses.

**Conclusions:**

Our systematic approach demonstrates that thousands of species may be impacted by disruption in one class of mutualisms, but extinctions will likely disrupt other mutualisms, as well. Although uncertainty is high, there is evidence that mutualism disruption directly threatens significant biodiversity in some geographic regions. Conservation measures with explicit focus on mutualistic functions could be necessary to bolster populations of widowed species and maintain ecosystem functions.

## Introduction

Driven by anthropogenic activities, current species losses are reaching mass extinction levels [1]. As species disappear from an ecosystem, the roles they play are lost, as well [2]. Even if they are otherwise resilient to anthropogenic change, extant species may be affected by disrupted interspecific interactions, losing their prey, predators, competitors, parasites, or mutualists.

Loss of ecological interactions can impact a wide array of ecosystem processes [3]. It has been argued that every species on Earth participates in one or more mutualisms (where mutualisms are defined as interactions with fitness benefits for both interacting partners) [4]. As such, broken ecological interactions may by themselves impact global biodiversity, by threatening species that are otherwise unaffected by major drivers of environmental change such as habitat loss, climate change, biological invasions, and overexploitation. Such species are thus vulnerable *because* their partners are vulnerable, not because they themselves respond directly to broad scale environmental change [5]. Species may be particularly affected by mutualism disruption because mutualisms are thought to evolve in response to stressful conditions or to help species overcome limiting factors (such as nutrient limitation, dispersal barriers, or predation) [6]–[8]. Declines in populations following mutualism loss have appeared in a growing number of case study organisms, such as vertebrate-dispersed trees in Peru [9], bird-pollinated plants in New Zealand and Hawaii [10], [11], and ant-tended trees in Africa [12].

In spite of the potential for mutualism disruption to impact biodiversity, there is only limited understanding of the quantitative scope of this threat and the magnitude of biodiversity that may currently be affected. An examination of tightly coevolved, host-affiliate relationships, including both mutualists and parasites, estimated that over 6000 species are in danger of extinction due to imminent host extinction [13]. However, we know of no studies that have attempted to quantify likely disruptions in the broader realm of mutualisms, including facultative and diffuse relationships (which represent the likely majority of mutualisms), or to project the impact of such disruptions on extant partner reproduction and/or survival.

Quantifying the abundance or vulnerability of mutualisms carries enormous challenges. Relative to antagonistic interactions, mutualisms have been historically understudied [14]. Systems known to host large mutualism diversity include areas in which new species continue to be discovered at a high rate, such as tropical rainforests and soils [15], [16]. The majority of species involved in mutualisms globally are likely microbes or invertebrates [17], [18], two groups whose diversity and extinction risk remain largely speculative [19], [20]. Assessments of mutualism disruption risk necessarily include quantification of the number of partners per mutualist, but this is known only for short-term case studies in particular study sites, and varies immensely [21].

With so many unknowns, quantifying the risk of mutualism disruption through a traditional meta-analysis or similar assessment remains elusive. However, examination of the scope of this problem is immediately important for global conservation efforts. Without consideration of mutualism disruption, assessments of extinction rates pegged to specific anthropogenic behaviors or following particular management decisions are likely to be substantially underestimated. For this reason, even assessing the order of magnitude of this threat may guide future research and enable effective decision-making for biodiversity conservation. We set out to evaluate global mutualism disruption risk for a particular class of mutualisms that has received a fair amount of research attention over the past century and for which such estimation is feasible: vertebrate-mediated plant reproductive mutualisms, specifically pollination and seed dispersal. Our examination of the risk of mutualism disruption for this group demonstrates the potentially broad scope of the problem and its implications for biodiversity.

The reproductive success of most flowering plant (angiosperm) species depends either wholly or partly on animal mutualists providing pollination, dispersal, or seed processing [22]. Animal extinctions disrupting these relationships may create “widow” plant species (*sensu* [23]) that exhibit reduced fitness and are vulnerable to coextinction [24], [25]. Using systematic review and synthesis of data from a wide diversity of studies, we here develop rough but quantitative estimates of the global number of angiosperm species facing widowhood due to the extinction of reproductive mutualist vertebrates. We also estimate the likely impact of widowhood on plant reproductive success. Although invertebrates represent the large majority of animal pollinators, we focus on vertebrate pollinators and seed dispersers because their geographic distributions and conservation status are sufficiently well characterized to quantify widowhood risks for plants associated with them. In stepwise fashion, we combine the following estimates: (a) the global number of vertebrate-pollinated and vertebrate-dispersed angiosperms; (b) the average number of vertebrate partners per plant species, both globally and by geographic region; (c) the proportion of such partners that are currently threatened with extinction, both globally and by geographic region; and (d) the average decline in reproductive success that widows are likely to experience. Even for vertebrate-plant mutualisms, which have a relatively broad scientific literature, quantification of the risk of disruptions requires synthesis of case studies and extrapolation from a few well-studied systems to derive global estimates. We recognize this limitation and the assumptions underlying our calculations and emphasize in our discussion both the order of magnitude of the estimates (rather than specific numbers) as well as the relatively highest-risk geographic regions. We argue that this first rough stab at quantification carries substantial heuristic value and believe it will spur crucial consideration of mutualism disruptions in ecological research and management.

## Methods

To approximate the global number of vertebrate-pollinated and vertebrate-dispersed plants, we gathered and combined the following published estimates: total global angiosperm species richness [26], the proportion of angiosperms that are animal-dispersed minus those that are ant-dispersed [7], [27], and the number of genera that are vertebrate-pollinated [27], [28]. Known instances of complete disruption of diffuse mutualisms (i.e., loss of the entire suite of pollinators or dispersers for plants that interact with multiple animal species) have occurred on oceanic islands [29]–[31], where the number of partners per plant is lower and partners more threatened than on continents. Therefore, we examined island (defined as sub-continental landmasses surrounded by water) and continental values separately. We estimated the number of vertebrate-pollinated and vertebrate-dispersed plants that are island endemics by deriving the percentage of total angiosperms that are island endemics from regional estimates of plant diversity [26] and assuming that the same percentage holds across our target classes.

We used a thorough literature search to develop a database of vertebrate pollinators and dispersers in order to assess global conservation threats to these guilds. The search was performed July-September 2010 in Web of Science and augmented by Google Scholar. The search covered the years 1899–2010 and included all combinations and derivations of the following terms: dispersal, pollination, frugivory, mutualism, vertebrate, mammal, bat, bird, lizard, tortoise, fish. Hundreds of resulting publications were examined for evidence of vertebrate participation in pollination or seed dispersal. Frugivory and granivory with the potential for seed dispersal is common among vertebrates, with fruit and seeds as ready resources to supplement diets that generally include or are dominated by other items [32]–[34]. Many more animals have been identified as frugivorous or granivorous than as seed dispersers, per se. We therefore additionally examined vertebrate ecology guides [35], [36] to identify species known to consume at least some fruits or to cache or drop some seeds, and we included these species in our seed disperser database. We reasoned that this made our assessment of extinction risk more conservative than if we had included only species for which seed dispersal itself has been documented, because fruit is consumed by so many generalist species, and those species are less likely to be threatened than are specialists [37]. Our search terms did not limit our seed disperser list to fleshy fruit dispersers: dry seeds may be cached by rodents, for example, or dispersed in the feces of ungulates, and we attempted to capture these behaviors, as well. To avoid underestimating the number of vertebrates involved, we also assumed that certain groups were involved in mutualisms: for example, we included all hummingbirds in our pollinator database and all thrushes in our seed dispersal database. We made such assumptions more for the seed disperser list than the pollinator list, since fruits and seeds are an easily-obtained resource evolved for availability and can therefore be at least secondary dietary components for a broad range of species (e.g., [38]). As long as such consumption has been identified for some members of a given animal genus (e.g., [39]) and no exceptions have been identified in published literature, we included that genus in the database. Finally, we included in our list of pollinator lizards all species in a database provided by J. Olesen, who has studied lizard pollination extensively [34], [40]; and in our seed disperser list all fish in a dataset provided by S. Correa, who published a recent review of fish as seed dispersers [41].

The resulting database is unlikely to be comprehensive: there may be publications not captured by our search terms, and many species that are frugivorous/granivorous may not be reported as such in published literature. For taxa for which we included all representatives (e.g., hummingbirds), our database is missing any species not included in the Red List. However, we expect that our database contains the majority of vertebrate pollinators and seed dispersers and is therefore an acceptable tool with which to estimate threat risk across these guilds. As long as missing species are randomly distributed across taxa and threat levels, they are unlikely to bias our threat level calculations. More likely to create bias are actual knowledge gaps, where certain rare taxa are less likely to be studied than others. However, in the context of our approach, such groups make our estimates more conservative (described further below).

We next extracted the species-specific conservation status of each organism in the database from the International Union for the Conservation of Nature (IUCN) Red List, version 2012.2. Developed and updated by the IUCN Species Programme, the Red List rates species as Least Concern, Near Threatened, Vulnerable, Endangered, Critically Endangered, Extinct in the Wild, and Extinct. These values are complex but quantitative: a ranking of Vulnerable, for example, is given when a species has shown or is predicted to show decline of 30% in a 10-year period and the causes of decline are irreversible, a 50% decline and the causes are reversible, or a substantially restricted range or population corresponding to specific criteria. A ranking of Endangered signifies that a species has shown or is predicted to show decline of 50% in a 10-year period and the causes of decline are irreversible, a 70% decline and the causes are reversible, or severely limited range corresponding to explicit criteria. A ranking of Critically Endangered indicates that a species has shown or is predicted to show decline of 80% in a 10-year period and the causes of decline are irreversible, a 90% decline and the causes are reversible, or range restrictions corresponding to Critically Endangered criteria (more detailed criterion descriptions available at www.iucnredlist.org). The category of Near Threatened is applied when a species has not yet declined enough to enter any of the other threat categories but shows known reduction and its future entry into a threat category is likely. Even many species considered “Least Concern” are identified as declining in Red List descriptions. For this study, however, all Least Concern species are considered non-threatened. Following the lead of similar threat assessments elsewhere [42], [43], we considered all species rated Near Threatened or worse to be “threatened” for the purpose of our calculations, since these show quantifiable decline. We removed extinct species from our database. For the small number (25) of bird and mammal species in our database that have not yet been evaluated by the IUCN, we conducted a literature review to determine whether there is any indication of notable decline. In the absence of such indication, we considered the species “Least Concern.” This was a conservative determination, given our goals, since such species may instead be understudied. For fish and reptiles, we found that a substantial proportion of known seed dispersers (72% and 59%, respectively) have not yet been evaluated by the IUCN. This highlights a pervasive lack of knowledge regarding the conservation status of these taxa. Because our methods treated non-evaluated and data-deficient species as Least Concern (i.e., non-threatened), it is likely that we underestimated threat rates among these taxa.

**Figure 1:**
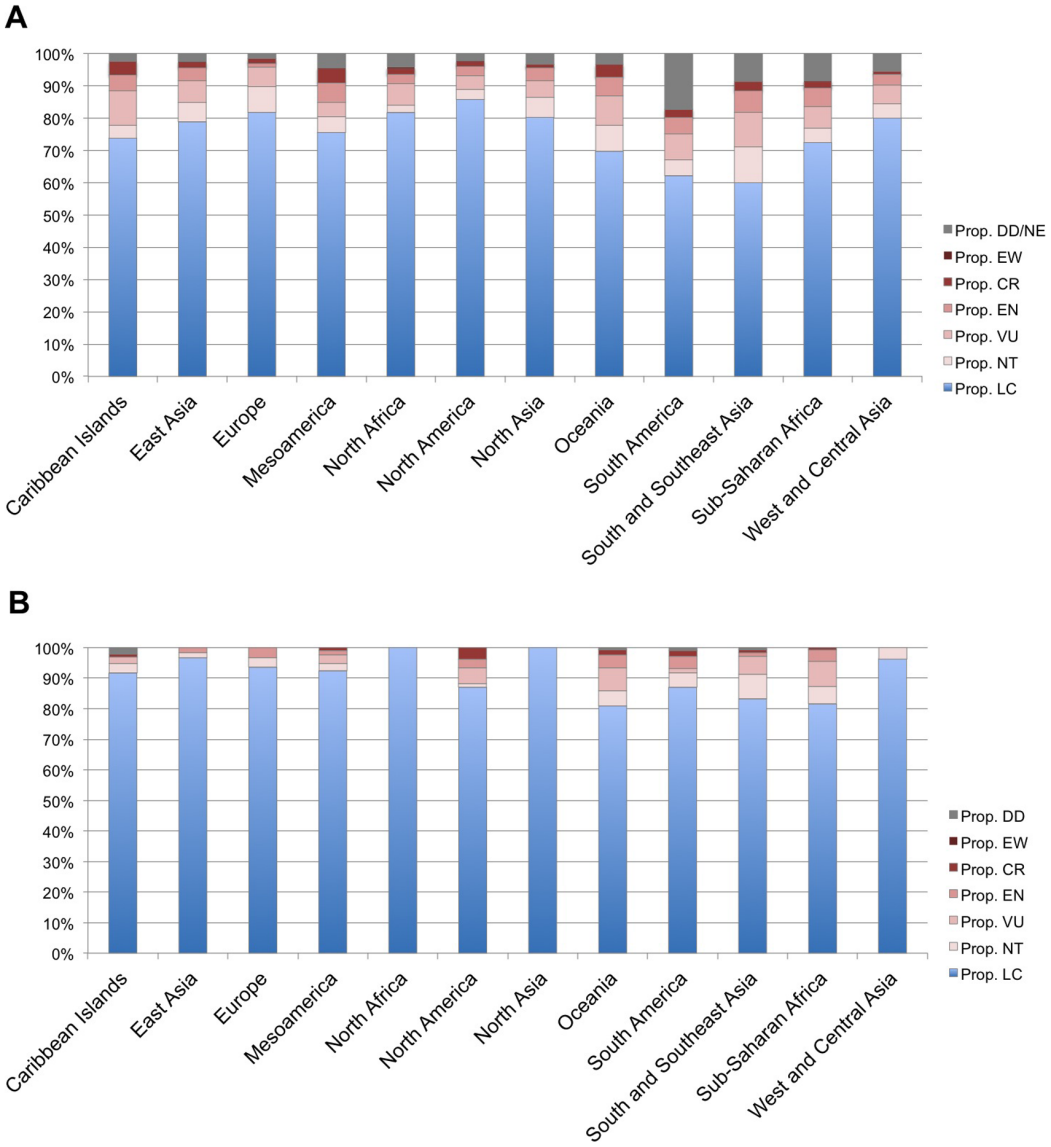
IUCN Red List threat levels by geographic region. **a**) Threat levels for vertebrate seed dispersers. **b**) Threat levels for vertebrate pollinators. Geographic regions are as provided in the IUCN Red List (www.iucnredlist.org). DD  =  Data Deficient. EW  =  Extinct in the Wild. CR  =  Critically Endangered. EN  =  Endangered. VU  =  Vulnerable. NT  =  Near Threatened. LC  =  Least Concern.

To calculate the proportion of plants likely to lose all of their vertebrate mutualists, we first obtained the geographic distributions of all vertebrate mutualists in our database from their records in the IUCN Red List, which assigns species to the geographic regions Antarctica, Caribbean Islands, East Asia, Europe, Mesoamerica, North Africa, North America, North Asia, Oceania, South America, South and Southeast Asia, Sub-Saharan Africa, and West and Central Asia (Fig. 1). We then developed independent calculations for these and broader geographic categories as outlined below. Additionally, we separately considered insular and mainland species because insular vertebrates have inherently smaller population sizes, increasing their probability of extinction compared to mainland species [44].

**Figure 2:**
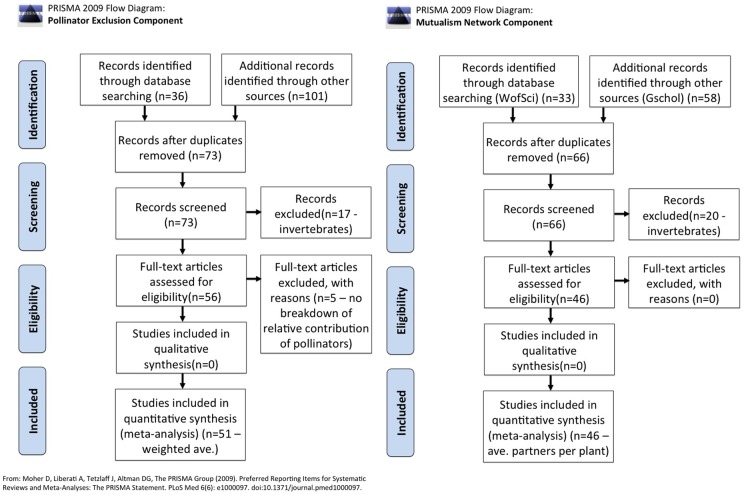
PRISMA flowchart providing the steps of data collection for this systematic review.

To estimate the number of partners per plant species, we first analyzed published comprehensive mutualism networks. Through a comprehensive literature search (in conformance with PRISMA (Preferred Reporting Items for Systematic Reviews and Meta-Analysis, www.prisma-statement.org) guidelines, search statistics are reported in Fig. 2), we identified 46 published interaction networks (28 dispersal and 18 pollination) that included vertebrate interactors and presented quantitative information on numbers of partners per species (Text S1). The sample size of such networks, which require a large amount of field observation, is unfortunately quite small, particularly for some geographic regions. We therefore performed a supplementary literature search using the terms *mammal*, *bird*, *bat*, *lizard*, *reptile*, and *fish*, each paired first with *pollination* and then with *seed dispersal*, in ISI Web of Science. This search netted additional plant-focused studies that identified the dispersers and pollinators of specific plants and included vertebrates as partners. Of resulting studies, we included in our analyses only those reporting number of partners for at least two plant species (a total of 26 studies). This criterion was applied in order to allow variance calculation, but it also served to exclude outliers (e.g., a plant selected for study because it is pollinated by only a single, highly-specialized partner). While each of these studies allowed calculation of number of partners per plant, this set of additional studies (Text S1) differed qualitatively from the network studies. Network studies examine a broad range of plants, many of which interact as much or more with invertebrate partners as with vertebrate partners, but tend to focus on a single geographic site. The supplementary plant-focused studies, by contrast, tended to focus on specific plant species that were often targeted by researchers *because* they are vertebrate-pollinated or –dispersed (e.g., studies interested in certain pollination syndromes). Furthermore, such studies frequently examined target plants across multiple study sites. As a result, the number of vertebrate partners per plant tended to be higher among these supplementary studies, but the dependence of the plants on their vertebrate partners is also likely higher because the plants are more specialized for vertebrate-mediated mutualisms (i.e., less likelihood of loss of all vertebrate partners, but greater consequences from such losses than would be expected among species interacting in a larger network including both invertebrate and vertebrate partners).

Considering pollination and dispersal studies separately, we calculated the average number of vertebrate partners per plant in each network or supplementary study. We then combined these values with global and regional threat levels to calculate rough estimates of the proportion of vertebrate-partnered plant species for which all partners are likely threatened. For example, because 26% of all seed dispersers are considered threatened, we assumed that there is a 26% chance that seed disperser A of a given plant is in that threatened group. Since plants in dispersal networks have an average of 6.00 seed dispersers, the same chance exists for seed dispersers B, C, D, E, and F. These probabilities are multiplicative, so the total chance that all of the seed dispersers for the plant are threatened becomes 0.26^6.00^. Importantly, this assumes an equal threat risk across all vertebrate partners (i.e., ignores species identity and assigns each species a generic, average rate). In reality, each plant likely partners with a variety of species, some of which are more generalist and some more specialized, and the chance of extinction likely varies among partners, as well. Certain regions (such as oceanic islands) and/or certain guilds (such as primates) exhibit particularly high threat rates; plants that occur in those regions or partner with those guilds likely face exorbitantly high risk of loss of all partners. Our approach, however, assumes that low extinction rates for some guilds are roughly offset by high rates for other guilds. Using our database of vertebrate mutualists and Red List categories, we calculated threat rates for dispersers and pollinators a) globally; b) by geographic region (if our set of networks and supplementary studies included at least two studies from the geographic region, enabling calculation of mean and standard error); c) by broader geographic regions (Old vs. New World, tropical vs. temperate, and hemispheres) selected because the set of studies contained sufficient sample size within each region to enable greater confidence in our results; and d) by islands vs. continents. To obtain island vs. continental values for pollinators, we examined each mutualist to determine if it was island-restricted or continental. For dispersers, since the database is much larger, we used random numbers generated in Microsoft Excel to sample a total of 1000 vertebrate seed dispersers, distributed proportionally among classes according to the proportions in which they occur in the full database (38.6% birds, 55.4% mammals, 2.5% reptiles, and 3.5% fish), and then identified each of these as island-restricted or not based on IUCN Red List distribution descriptions.

The limited availability of key information inhibited our calculation of confidence intervals for our estimates. The three primary information sources we used to estimate the number of plants likely to be impacted were: published estimates of the diversity of plant species, the proportion of known vertebrate pollinators and dispersers that are threatened, and the average number of vertebrate partners per plant. Only one of these (average number of partners per plant) allowed variance calculations, and that variance was computed from interaction networks and supplementary studies. The threatened proportion of pollinators and dispersers was a direct count of all vertebrate mutualists ranked as threatened in the IUCN database, so that value has no variance. Published estimates of overall diversity of plant species and of diversity of vertebrate-mutualist plant species are provided as point estimates, with no associated variance, in the literature [7], [26]–[28]. The confidence intervals we calculated are therefore based on partial variance and do not take into account uncertainty in total diversity of vertebrate-mutualist plants. For this reason, we emphasize in our results and discussion the percent of regional plants affected, rather than the estimated count. As global estimates of plant diversity rise, the numerical estimate of plants affected by vertebrate mutualist loss will rise as well, while the percentage remains constant.

**Figure 3:**
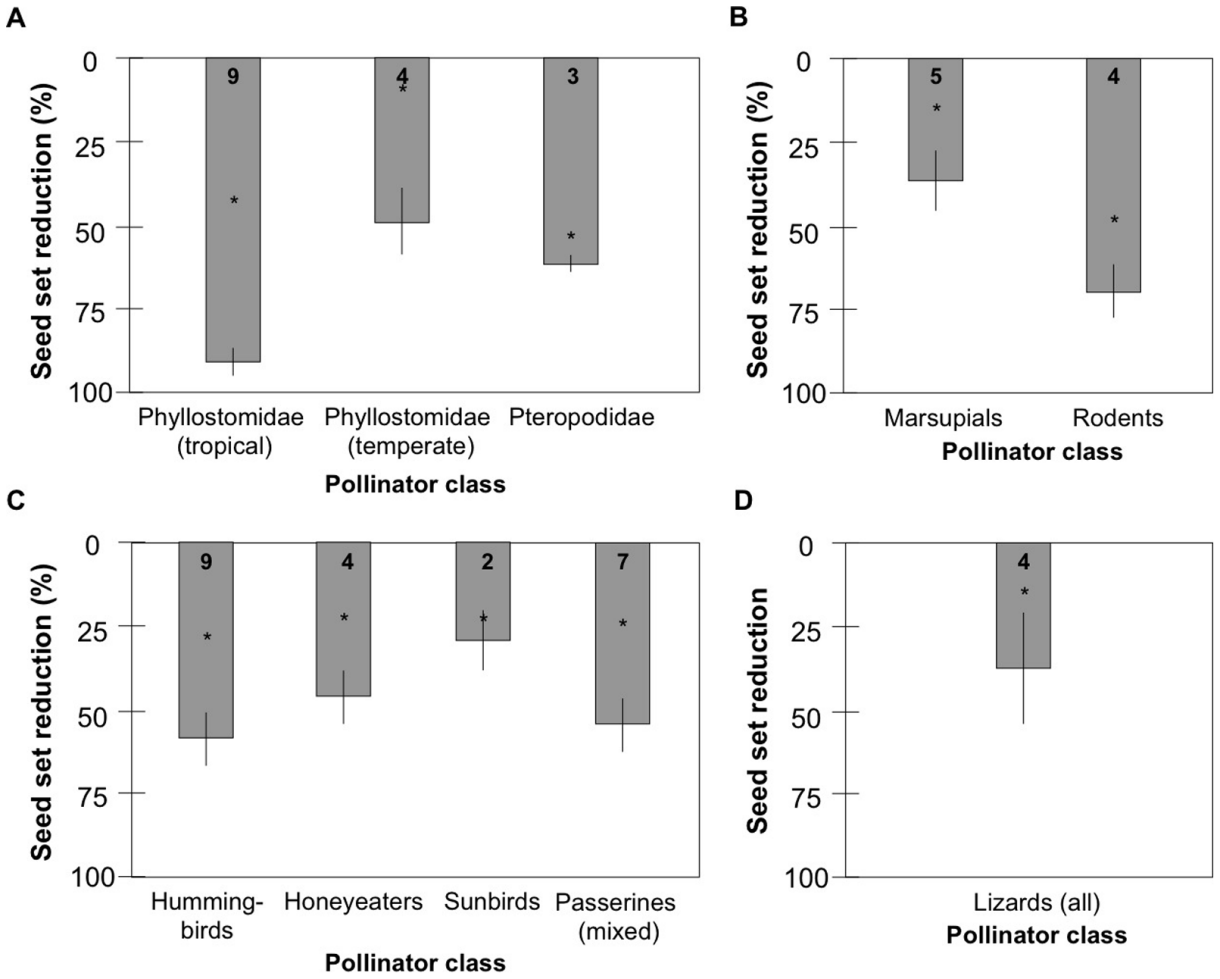
A review of pollinator exclusion studies yielded weighted mean seed set reductions for widows previously pollinated by different vertebrate classes. **a**) Seed set reductions after bat exclusion for bat-pollinated plants. **b**) Seed set reductions after nonvolant mammal exclusion for nonvolant mammal-pollinated plants. **c**) Seed set reductions after bird exclusion for bird-pollinated plants. **d**) Seed set reductions after lizard exclusion for lizard-pollinated plants. Bars depict weighted means. Vertical lines represent standard error. The minimum seed set observed in any exclusion study is indicated with an asterisk (*); this is the minimum predicted effect of loss of vertebrate pollinators for a given plant species. Bold numbers at the top of each bar provide the number of independent publications used to calculate each weighted mean.

The observed and estimated impact of mutualism disruption on plant reproduction varies by species. We conducted a comprehensive literature review of vertebrate pollinator exclusion studies to estimate the average seed set reduction likely to result from extinction of all vertebrate pollinators of any given angiosperm species (Figs. 2,3; Text S1). These studies had quantified the percent of seed set attributable to specific vertebrate classes by excluding that class but not other potential pollinators (e.g., invertebrates). Our use of these studies enabled us to separate the proportion of seed set generated by vertebrate pollination from seed set attributable to self-fertilization and invertebrates. For each vertebrate class, we calculated an average, weighted by sample size, of the percentage reduction in seed set across all studies relevant to that class. The results of these weighted averages are midrange estimates of seed set reduction in the absence of each vertebrate class (Fig. 3).

When vertebrate dispersal is lost, plants may experience reduced reproduction for two main reasons: first, because vertebrate gut processing often enhances seed germination [45], and second, because dispersal away from the parent plant can remove offspring from density dependent pathogens and competition and results in elevated seedling survival [46]. We discuss implications of disruptions in these processes in the context of previously-published meta-analyses examining them [46], [47].

## Results

Based on our literature review of published expert estimates, we assumed a total global angiosperm species richness of 300,000 species [26]. The proportion of angiosperm species that are animal-dispersed was estimated from published literature at 0.56 [27], for an estimated richness of 0.56*300000 = 168,000. Given a published expert estimate of 11,000 angiosperm species that are ant-dispersed [7], the difference between these groups should approximate the number of vertebrate-dispersed species: 168,000–11,000 = 157,000 = 52.3%. Our literature-derived estimate for the proportion of genera that are vertebrate-pollinated was 0.056 [28]. This number likely underestimates the true total number because it is based solely on bird- and bat-mediated pollination, but it is the sole available estimate we have found. If the same proportion holds across species, as well, we can estimate that there are approximately 300,000*0.056 = 16,800 vertebrate-pollinated species. Finally, our literature-derived estimate of the proportion of angiosperms that are restricted to islands was 25.9% [26]. If the same proportion holds across our target classes, we can roughly estimate that islands contain 44,988 vertebrate-pollinated and vertebrate-dispersed plant species (total angiosperms * proportion on islands * (proportion animal-dispersed + proportion animal-pollinated)  = 300,000 * 0.259 * (0.523+0.056)).

Taking the above values in combination with our vertebrate pollinator and disperser datasets (Dataset S1), we estimate that approximately 16,800 plant species are vertebrate-pollinated and 157,000 angiosperms vertebrate-dispersed by at least 1162 vertebrate pollinators and 6782 vertebrate dispersers, respectively. Of these vertebrate mutualists, globally, 16.5% of pollinators (192 species) and 25.9% of dispersers (1758 species) are currently threatened with extinction [48]. Threat levels are particularly high for island-based species in our database: we estimate that 30.4% of island-based vertebrate pollinators are threatened, while 40.2% of island-based vertebrate seed dispersers are threatened.

Calculating from characterized mutualism networks and supplementary studies identifying partners of focal plants, we estimate that, globally, vertebrate-pollinated plants are pollinated by an average of 2.45 vertebrates per plant species, while vertebrate-dispersed plant species are dispersed by an average of 6.00 partners. Considering island and continental species separately and integrating these values with known vertebrate threat levels (e.g., for islands, 30.4% of pollinators are threatened and plants are pollinated by an average of 2.08 partners, so the proportion of island plants at risk of losing all vertebrate pollinators is calculated as 0.304^2.08^), we estimate that 8.4% of vertebrate-pollinated island plants or 365 (95% CI from 259 to 516) island plant species will lose all vertebrate pollinators if currently threatened vertebrates become extinct. Similar calculations for continental species, where networks are larger and extinction threats fewer, resulted in an estimated 44 (95% CI from 20 to 93) species in danger of complete vertebrate pollinator loss (Table 1). Once again, these species may be pollinated by additional, non-vertebrate means in addition to their vertebrate partners.

**Table 1:** Constituent values used to estimate the number of plants likely to be widowed if currently threatened vertebrate species become extinct.

Estimate (see text for methods and sources)*	Islands	Continents	Both
Total no. angiosperm species	77,700	222,300	
Percent total that are vertebrate-pollinated			5.6
Percent of total that are vertebrate-dispersed			52.3
No. vertebrate-pollinated species = A	4,351	12,449	
Percent vertebrate pollinators threatened = X	30.4	11.8	
No. partners per vertebrate-pollinated spp = L (±SE)	2.08±0.29	2.64±0.35	
No. vert-poll spp threatened with widowhood = A*(X^L^)	365	44	
No. vertebrate-dispersed species = B	40,637	116,263	
Percent vertebrate dispersers threatened = Y	40.2	22.1	
No. partners per vertebrate-dispersed spp = M (±SE)	2.94±0.39	7.36±1.02	
No. vert-disp spp threatened with widowhood = B*(Y^M^)	2788	2	
Total species threatened with loss of all vertebrate mutualists	3,153	46	
Sum of species threatened with loss of all vert. mutualists (global estimate)			
**Percent total angiosperm species threatened with loss of vert. mutualists:**			**1.1**

Estimates are extrapolated from well-known systems, focusing on islands and continents, so should be considered rough. Nevertheless, they are intended to allow assessment of the order of magnitude of potential mutualism disruption.

* All species richness estimates are rounded to integer values in order to be realistic.

Performing the same calculations for seed dispersers (Table 1) and then combining these estimates for islands and continents, our full estimate of plants currently at risk of losing all of their vertebrate mutualist partners becomes 3,199 species (95% CI from 2233 to 4595), or 1.1% of all angiosperm species (assuming a global total of 300,000 angiosperm species, intermediate among available estimates [7], [26], [28]) (Table 1). This result is driven largely by island species. When each mutualism is considered separately, 2.4% of all vertebrate-pollinated angiosperm species are at risk of loss of their vertebrate partners, while only 1.8% of vertebrate-dispersed angiosperm species face the same risk. On islands alone, where networks are more clearly defined and bounded by the limited geographies of the islands themselves, enabling our confidence in these estimates to be higher, the estimate of plants at risk of vertebrate widowhood is 3,153 (95% CI from 2213 to 4494), or 4.1% of island angiosperm species.

By zooming in to examine particular geographic regions, our approach can highlight those areas where mutualism disruption (i.e., loss of all vertebrate partners) is a particular risk. At the same time, the total number of plants for which all vertebrate partners have been identified in any particular geographic region is small, reducing the confidence of calculations. Some regions used in the IUCN Red List, for example, are represented by zero or a single study from which we could calculate number of partners per plant, and estimation of mutualism disruption risk for those regions is either impossible or highly uncertain (Table 2). Greater certainty can be obtained by combining IUCN regions to examine broader geographic areas, such as tropical vs. temperate regions; Old vs. New World; and hemispheres. Examining those geographic areas represented by at least two studies, our methods predict particularly high percentages of angiosperm species at risk of losing all vertebrate pollinators in Africa (4.2%), the Caribbean (2.1%), the Old World (3.5%), the northeastern hemisphere (2.8%), and the southeastern hemisphere (2.4%) (Table 2). Because number of partners per plant species is higher in seed dispersal mutualisms, likely buffering them from disruption, our methods predict high percentages of angiosperm species at risk of losing all vertebrate seed dispersers only in the Caribbean (2.1%) and Asia (3.1%).

**Table 2:** Average number of vertebrate partners per plant (P), proportion of vertebrate partners that are threatened with extinction (T), and proportion of total vertebrate-mutualist plants at risk of losing all vertebrate partners (T^P^), by geographic region.

	Pollinators				Seed Dispersers			
Geographic region	P (± SE)	N	T	T^P^ (± SE)	P (± SE)	N	T	T^P^ (± SE)
**IUCN Regions:**								
Caribbean Islands	1.40±0.06	3	0.063	0.021±0.003†	2.50±0.29	4	0.21	0.021±0.010†
Asia	1.24	1	0.12	0.073	2.50±1.50	2	0.25	0.031±0.12†
Europe	N/A	0	0.065	N/A	5.02±0.54	9	0.16	0.00012±0.0001
Mesoamerica	4.69±0.90	6	0.076	5.77×10^−6^±2.90×10^−5^	9.52±2.61	5	0.20	1.95×10^−7^±6.79×10^−6^
Africa	1.76±0.31	9	0.16	0.042±0.022†	7.62±2.02	4	0.18	2.34×10^−6^±3.66×10^−5^
North America	2.20±0.61	3	0.13	0.011±0.018	6.21±1.21	2	0.12	1.80×10^−6^±1.66×10^−5^
Oceania	2.97±0.60	5	0.18	0.0065±0.008	11.00±9.00	2	0.26	4.17×10^−7^±0.035
South America	2.08±0.35	13	0.12	0.012±0.010	4.85±0.30	4	0.20	0.00044±0.0002
**Tropical**	2.52±0.30	24	0.14	0.0065±0.004	6.66±1.23	19	0.24	7.28×10^−5^±0.0002
**Temperate**	2.34±0.46	16	0.066	0.0017±0.003	5.04±0.54	13	0.18	0.00016±0.0002
**Old World** (Europe, Asia, Africa)	1.66±0.23	11	0.13	0.035±0.017†	6.29±1.14	16	0.23	8.76×10^−5^±0.0002
**New World** (the Americas)	2.64±0.36	25	0.10	0.0025±0.003	5.96±1.11	15	0.19	5.20×10^−5^±0.0002
**Hemispheres:**								
Northeast	1.62±0.38	2	0.11	0.028±0.027†	4.62±0.63	10	0.24	0.0014±0.001
Northwest	3.24±0.63	12	0.084	0.00032±0.0007	6.37±1.51	11	0.18	1.60×10^−5^±0.0001
Southeast	2.21±0.33	13	0.18	0.024±0.014†	8.07±2.42	7	0.22	4.63×10^−6^±9.21×10^−5^
Southwest	2.08±0.35	13	0.14	0.018±0.013	4.85±0.30	4	0.22	0.00071±0.0003

Geographic affinities are derived from the IUCN Red List; subregions of Asia and Africa were combined into continental estimates due to low sample size of available studies providing number of partners per plant. N  =  number of studies available for each geographic region. †Regions with notably high risk (>2%). Note: where only a single study was available, N  = 1 and no variance calculation is possible. Such cases were omitted from discussion of high-risk regions. N/A  =  no studies available.

Dividing vertebrate pollinator exclusion studies by vertebrate group, minimum seed set loss resulting from exclusion of each group varied from 10–80%. Remaining seed set was attributable to invertebrate pollination or self-pollination. The weighted mean of seed set loss was 58.2±1.0% (mean ± SE) (Fig. 3), including cases of obligate, one-to-one mutualism as well as diffuse and facultative mutualisms.

Lost reproduction arising from dispersal failure ranges from 100% for species entirely dependent on vertebrate processing for germination(e.g., [49]), to 0% for species able to readily disperse by water or other media and to germinate without gut passage. Published meta-analyses provide estimates of the effects of vertebrate gut processing on seed germination (across all disperser taxa, germination was bolstered by an average of 28.4% following gut processing [45]) and the survival benefit to seedlings of removal from parental neighborhood (on average, seedling survival was boosted 31.6% by removal from parental neighborhood [50]). Together, these numbers suggest an estimated average decline in reproductive success of 40.6% after loss of all vertebrate dispersers associated with any given plant species.

## Discussion

While uncertainties are large, our extrapolative approach led to an estimate that extinctions of currently-threatened animal species may remove all pollination or seed dispersal services from thousands of angiosperm species, globally. As additional animals become threatened over time, the number of plant species affected will also rise. Because mutualism disruption has been so little explored empirically, our estimates are intended to elucidate the order of magnitude of the problem and to stimulate further research and discussion and are necessarily based on incomplete information and a series of assumptions.

There are at least three reasons that our numerical estimates may be conservative. First, we chose to focus on widowhood, or loss of all vertebrate (or invertebrate) partners, as the most conservative approach to estimating the number of plant species affected. Loss of some partners from a diffuse mutualism may also reduce extant partner species' fitness if remnant species act as partial seed predators, move seed/pollen shorter distances than did extinct partners (for example, rodents performing dispersal previously done by birds (e.g., [51])), or fail to numerically compensate for missing mutualists, but our analysis does not include such cases. Second, even when a pollinator or disperser species is not globally extinct, its extirpation from portions of its range can remove mutualist functions from more range-restricted plants. For example, the honeyeater *Myzomela rubratra* is considered “Least Concern” by the IUCN but has been extirpated from the island of Guam. Its absence has left the plant *Bruguiera gymnorrhiza*, native to Guam, without its primary pollinator [52]. Seed set for *B. gymnorrhiza* is now significantly lower in Guam than elsewhere [52]. Globally, estimated range contractions that have already occurred for declining mammals average 50% [53]. Range contractions for butterfly species in Spain have averaged one-third [54]. For comparison, models predict high threat levels across more than half of the distributions of evaluated amphibian species [55]. For birds, average range contractions of approximately one-quarter globally are projected by the year 2050 [56], and range contractions of over 50% are predicted for montane bird species by the year 2100 [57]. Predicted range shifts of tropical plants and invertebrates in Costa Rica suggest that half of examined species will likely vacate lowland areas and move to higher elevations [58]. Plant species affected by local extirpations of their mutualists are not captured by our approach but would increase the number of plants affected by mutualism disruption. Even strong reduction in regional mutualist numbers, without total extirpation, can have an effect if the mutualist becomes “ecologically extinct.” Under this scenario, the mutualist's numbers have decreased sufficiently that it has become functionally absent from the ecosystem [59]. Notably, ecological extinction may occur long before a species is completely absent [60]. Third, 15% of mutualist mammal species, 19% of reptiles, 23% of fish, and 0.6% of birds are listed in the IUCN database as “Data Deficient” [48] because their population trends are unknown. We included none of these in our assessment of threat rates for vertebrate mutualists. Since many of these understudied taxa are likely in decline [61], the vulnerability of mutualisms involving them may be higher than we have estimated.

By contrast to these considerations, our method could have generated overestimates due to one key metric: the average number of partners per plant species. This overestimate is possible because the sole line of evidence available for this value was the set of existing studies that have identified vertebrate partners for focal plants and networks. Such studies are of variable lengths, but most are fairly short-term (one to three seasons). However, a longer-term study in Greece, based on four continuous years of plant-pollinator interaction records, concludes that year-to-year turnover in interspecific relationships is high and that pollinator specialization may therefore be overestimated in many networks [21]. Since a larger number of partners per species resulted in a smaller estimate of widowhood risk in our calculations, larger-scale or longer-term evaluations might have detected larger numbers of partners per plant species, which would have reduced our overall risk estimate.

The consequences of mutualism disruption may vary widely from species to species and from region to region. Mutualism occurrence may vary across latitudinal gradients; for example, invertebrate-mediated pollination is proportionally more important at higher latitudes relevant to vertebrate-mediated pollination [62]. Widespread species may partner with more specialized or more threatened species in some portions of their range. Several species of columnar cacti specialize on bat pollinators in the tropical portions of their ranges, for instance, but use a more diffuse array of bat and bird pollinators at temperate latitudes [63]. As threatened vertebrates are lost, such plants may become widowed in portions of their ranges: for example, nearly all pollination of *Neobuxbaumia tetetzo* in tropical Mexican deserts is performed by the bats *Leptonycteris curasoae* and *Leptonycteris nivalis* [64]. According to the IUCN Red List, these bats are Vulnerable and Endangered, respectively, elevating the likelihood that cacti in the region will lose these specialist pollinators. To accurately assess risks of widowhood for a particular region and particular class of mutualisms, it will be necessary to examine regional mutualism networks and specific population trends.

If they become widowed, some plant species may partner with new pollinators or seed dispersers, forming novel mutualisms with non-native species or with native species that adopt the functional roles of extinct mutualists. For example, rodents and livestock currently disperse some plant species that were likely dispersed in the past by now-extinct megafauna [65], [66]. Novel mutualists may not emulate extinct mutualists perfectly, however [67]; partner shifts could lead to changes in plant population distributions, densities, and genetic structure [66]. Overall, general lack of information makes it difficult to precisely evaluate the global implications of mutualism disruption. Mutualisms have been carefully studied for only a small proportion of taxa, so the contributions of mutualisms to fitness can only be estimated. Our review of vertebrate exclusion studies suggests that plants are likely to experience substantial reductions in seed set if their vertebrate pollinators are lost, but continued pollination via selfing and invertebrate pollinators will retain some reproduction for most species. Among vertebrate-dispersed species, removal from the parental neighborhood via water or gravity remain possible following loss of animal dispersers, but abiotic dispersal may be more constrained by topography and microsite conditions than is biotic dispersal (i.e., biotic dispersal may be necessary for seeds to move uphill and long distances in non-aquatic environments) [67], [68]. For those plant species that do experience seed set reductions, reduced seedling survivorship, or lost gut processing, the consequences for fitness are unclear. For some species, reproductive declines may trigger population trajectories leading toward extinction [e.g., 69,70]. For many other species, adult survival is more important and changes in reproductive output exert smaller effects [71]. Population dynamics are highly species-specific, making generalization difficult. Furthermore, reproductive declines in the context of global environmental change could have impacts on population trajectories that are difficult to predict.

**Figure 4:**
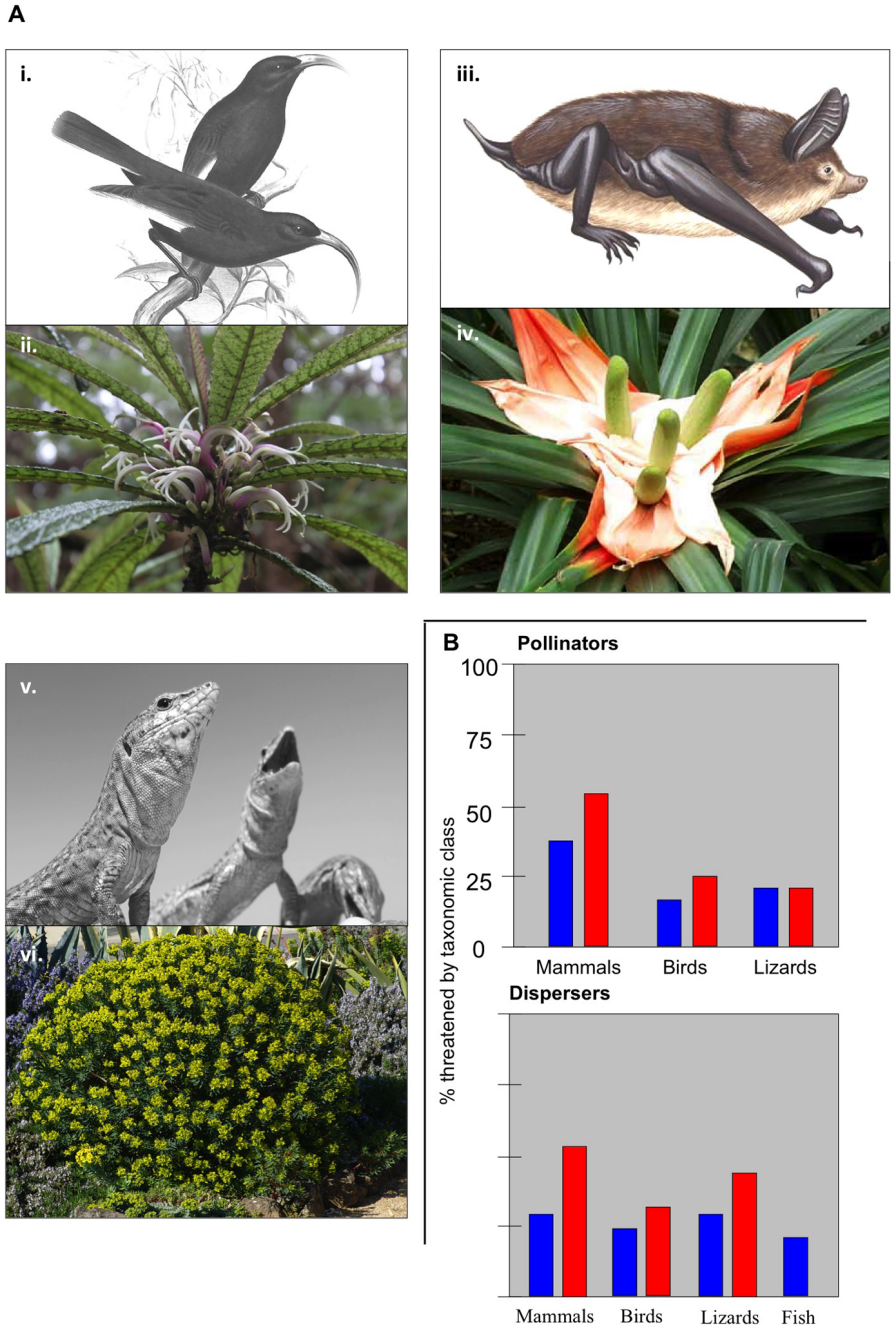
Existing widow plants demonstrate reduced reproduction, while threatened vertebrates suggest that many more species may become widowed, especially on islands. **a**) The extinction of honeycreepers including (i) Hawaii's black mamo (*Drepanis funerea*) resulted in widowhood for lobelioids including (ii) *Cyanea stictophylla* [29]. The near extinction of (iii) New Zealand's greater short-tailed bat (*Mystacina robusta*) widowed (iv) *Freycinetia baueriana* [30]. The island-scale extinction of (v) the lizard *Podarcis lilfordi* in the Balearic Islands disrupted the pollination of (vi) *Euphorbia dendroides* [31]. **b**) IUCN conservation status rankings for vertebrate pollinators and dispersers reveal that island endemic species (red bars) are more vulnerable by percent threatened than are vertebrate mutualists globally (green bars). Photo/image credits: F. W. Frowahk (*Drepanis funerea*); C. Aslan (*Cyanea stictophylla*); B. Duperron (*Mystacina robusta*); Armchair Travel and Kew Gardens (*Freycinetia baueriana*); D. André (*Podarcis lilfordi*); K. Kozminski (*Euphorbia dendroides*).

Mutualism disruption has been documented in certain systems, providing a glimpse of its likely implications. Ongoing declines in animal-pollinated or -dispersed plants have been linked to concurrent loss of mutualist animals in Central Africa [72], Tonga [73], and Australia [74], among other locations. These impacts are evident in spite of the higher number of partners per plant that can be expected in continental systems, providing further evidence that mutualism disruption is of global concern. Indeed, the existing data available for our analysis, though limited, imply that the eastern hemisphere (Asia, Africa, and Oceania) as a whole faces particularly high risk of mutualism disruption. Perhaps more intuitively, the “extinction debt” on oceanic islands in particular may be considerable: declines resulting from lost mutualisms, even when leading inevitably to extinction, are likely to be slow because plants are long-lived and many self-pollinate to some degree [75]. A wave of widowhood-induced plant species extinctions appears increasingly likely following the animal extinction pulse driven by European colonization of the world's islands [75], [76]. Recorded reductions in vertebrate-pollinated plant populations on islands are consistent with this hypothesis (Fig. 4) [77], [78].

Widowhood will likely also interact with other drivers of rapid environmental change to increase the vulnerability of widows to coextinction. In the coming decades, more and more species will be affected by habitat loss and climate change [79]. Interruptions of dispersal and pollen transfer may compound the impacts of these environmental stressors by reducing the ability of plant species to adapt and migrate in response to changing conditions. While uncertainty remains high, the evidence we have assembled here suggests that mutualism disruption is a global risk that crosses habitats and taxa and may have substantial impact on widow species population trajectories. Additional questions that must be addressed by the scientific community in order to evaluate the scope of this threat include: What are the likely long-term fitness consequences of mutualism disruption, across mutualism types and taxa? Under what circumstances might non-native species partner with widowed native species, and with what consequences for native communities? What are the evolutionary implications of broken mutualisms for widowed species? What factors promote or hinder functional redundancy among potential mutualists (i.e., when and how well can mutualists compensate for one another)? If the data assembled here reflect broader patterns with accuracy and thousands of plant species face potential widowhood, close examination and refinement of potential remedies should be a high priority. Explicit incorporation of mutualisms into conservation assessments, as well as direct mutualism restorations, may significantly bolster the success of biodiversity protection measures [80].

## Supporting Information

Text S1 **Sources used in quantitative estimates.**
(DOC)

Text S2 **PRISMA Checklist for systematic review.**
(DOC)

Dataset S1 **Vertebrate seed dispersers and pollinators.**
(XLS)
