# Supplementary material for: Mutualism Disruption Threatens Global Plant Biodiversity: A Systematic Review
Source: PLoS One. 2013 Jun 19;8(6):e66993. doi: 10.1371/journal.pone.0066993 (PMC3686776; doi:10.1371/journal.pone.0066993)
Supplement: Text S1 — Sources used in quantitative estimates. (DOC) [file pone.0066993.s001.doc]

**Supplementary Information**

**Table S1.** Mutualistic network and supplementary plant-focused studies used to calculate the proportion of plants with single vertebrate mutualist partners and the average number of vertebrate partners per plant in diffuse mutualisms. Locations in bold are temperate; remaining locations are tropical.

| Mutualism Type | Island | | Continental | |
| --- | --- | --- | --- | --- |
|  | Location | Citation | Location | Citation |
| Pollination | Jamaica | 1 | **Arizona, USA** | 15 |
|  | Borneo | 2 | Malaysia | 16 |
|  | **Chiloé Island** | 3 | Brazil | 17 |
|  | Trinidad | 4 | Venezuela | 18 |
|  | **Canary Islands** | 5 | Costa Rica | 19 |
|  | Mauritius | 6 | **Argentina** | 20 |
|  | Ile aux Aigrettes | 7 | Costa Rica | 21 |
|  | **Tasmania** | 8 | Venezuela | 22 |
|  | Antilles | 9 | **Western Australia** | 23 |
|  | **Juan Fernández** **Islands** | 10 | **South Africa** | 24 |
|  | **New Zealand** | 11 | **Mexico** | 25 |
|  | Madagascar | 12 | Andes | 26 |
|  | African islands | 13 | **Georgia** | 27 |
|  | West Indies | 14 | Brazil | 28 |
|  |  |  | **Arizona** | 29 |
|  |  |  | **South Africa** | 30 |
|  |  |  | Nigeria | 31 |
|  |  |  | **South Africa** | 32 |
|  |  |  | Mexico | 33 |
|  |  |  | Venezuela | 34 |
|  |  |  | Mexico | 35 |
|  |  |  | Brazil | 36 |
|  |  |  | **Argentina** | 37 |
|  |  |  |  |  |
| Dispersal | Puerto Rico | 38 | **New Jersey, USA** | 46 |
|  | Philippines | 39 | **South** **Africa** | 47 |
|  | Papua New Guinea | 40 | **Spain** | 48 |
|  | **Japan** | 41 | Brazil | 49 |
|  | **England** | 42 | Various (source of several) | 50 |
|  | Trinidad | 43 | Various (source of several) | 51 |
|  | **England** | 44 | **Spain** | 52 |
|  | Madagascar | 45 | Mexico | 53 |
|  |  |  | Malaysia | 54 |
|  |  |  | **China** | 55 |
|  |  |  | Kenya | 56 |
|  |  |  | Brazil | 57 |
|  |  |  | Costa Rica | 58 |
|  |  |  | Panama | 59 |
|  |  |  | Kenya | 60 |
|  |  |  | Costa Rica | 61 |
|  |  |  | **Mediterranean** | 62 |
|  |  |  | **Illinois** | 63 |
|  |  |  | **Mediterranean** | 64 |

**Table S2.** Studies used to estimate likely seed set reductions in the absence of each vertebrate pollinator class. N provides the sample size used to calculate weighted means. Where multiple N’s are provided, study reported results for multiple plant species.

| Vertebrate class | Citation | N | |
| --- | --- | --- | --- |
| Hummingbird | 65 | 87 | |
| Hummingbird | 66 | 31, 18 | |
| Hummingbird | 67 | 30 | |
| Hummingbird | 68 | 98 | |
| Hummingbird | 69 | 138, 60 | |
| Hummingbird | 70 | 20 | |
| Hummingbird | 71 | 88 | |
| Hummingbird | 72 | 37 | |
| Hummingbird | 73 | 60 | |
| Honeyeater | 74 | 38 | |
| Honeyeater | 75 | 15 | |
| Honeyeater | 76 | 35 | |
| Honeyeater | 77 | 3 | |
| Passerines (general) | 78 | 10, 10, 12, 14, 10 | |
| Passerines (general) | 79 | 19 | |
| Passerines (general) | 80 | 30 | |
| Passerines (general) | 81 | 9 | |
| Passerines (general) | 82 | 10 | |
| Passerines (general) | 83 | 150 | |
| Passerines (general) | 84 | 30 | |
| Sunbirds | 85 | 7 | |
| Sunbirds | 86 | 82 | |
| Rodents | 87 | 13 | |
| Rodents | 88 | 9, 27 | |
| Rodents | 89 | 23 | |
| Rodents | 90 | 20 | |
| Marsupials | 74 | 43 | |
| Marsupials | 91 | 13 | |
| Marsupials | 92 | 8 | |
| Marsupials | 93 | 131 | |
| Marsupials | 94 | 16 | |
| Pteropodidae | 95 | 20 | |
| Pteropodidae | 96 | 20 | |
| Pteropodidae | 97 | 28, 4 | |
| Phyllostomidae – tropical | 98 | 70 | |
| Phyllostomidae – tropical | 99 | 28, 30 | |
| Phyllostomidae – tropical | 100 | 25 | |
| Phyllostomidae – tropical | 101 | 223 | |
| Phyllostomidae – tropical | 102 | 40 | |
| Phyllostomidae – tropical | 103 | 32 | |
| Phyllostomidae – tropical | 104 | 15 | |
| Phyllostomidae – tropical | 105 | 34, 38, 35, 31 | |
| Phyllostomidae – tropical | 72 | 13 | |
| Phyllostomidae – extratropical | 106 | 22 | |
| Phyllostomidae – extratropical | 71 | 154 | |
| Phyllostomidae – extratropical | 107 | 100, 62, 79, 180 | |
| Phyllostomidae – extratropical | 65 | 83 | |
| Lizards | 108 | 18 | |
| Lizards | 84 | 30 | |
| Lizards | 109 | 13 | |
| Lizards | 110 | 5 |  |

**Supplementary References**

1. Percival M. (1974) Floral ecology of coastal scrub in southeast Jamaica. Biotropica 6: 104-129.

2. Sakai S, Kato M, Inoue T (1999) Three pollination guilds and variation in floral characteristics of Bornean gingers (Zingiberaceae and Costaceae). Am J Bot 86: 646-658.

3. Smith-Ramírez C, Martinez P, Nuñez M, González C, Armesto JJ (2005) Diversity, flower visitation frequency and generalism of pollinators in temperate rain forests of Chiloé Island, Chile. Bot J Linn Soc 147: 399-416.

4. Snow BK, Snow DW (1972) Feeding niches of hummingbirds in a Trinidad valley. J Anim Ecol 41: 471-485.

5. Dupont YL, Hansen DM, Olesen JM (2003) Structure of a plant-flower-visitor network in the high-altitude sub-alpine desert of Tenerife, Canary Islands. Ecography 26: 301-310.

6. Kaiser-Bunbury CN, Memmott J, Müller CB (2009) Community structure of pollination webs of Mauritian heathland habitats. Perspect Plant Ecol 11: 241-254.

7. Oleson JM, Eskildsen LI, Venkatasamy S (2002) Invasion of pollination networks on oceanic islands: importance of invader complexes and endemic super generalists. Divers Distrib 8: 181-192.

8. Hingston AB, McQuillan PB (2000) Are pollination syndromes useful predictors of floral visitors in Tasmania? Austral Ecol 25: 600-609.

9. Martén-Rodríguez S, Almarales-Castro A, Fenster CB (2009) Evaluation of pollination syndromes in Antillean Gesneriaceae: evidence for bat, hummingbird and generalized flowers. J Ecol 97: 348-359.

10. Anderson GJ, Bernardello G, Stuessy TF, Crawford DJ (2001) Breeding system and pollination of selected plants endemic to Juan Fernández Islands. Am J Bot 88: 220-233.

11. Anderson SH (2003) The relative importance of birds and insects as pollinators of the New Zealand flora. NZ J Ecol 27: 83-94.

12. Baum DA (1995) The comparative pollination and floral biology of baobabs (Adansonia-Bombacaceae). Ann Miss Bot Gard 82: 322-348.

13. Olesen JM, Alarcón M, Ehlers BK, Aldasoro JJ, Roquet C (2012) Pollination, biogeography and phylogeny of oceanic island bellflowers (Campanulaceae). Persp Plant Ecol Evol Syst 14: 169-182.

14. Dalsgaard B, Martín González AM, Olesen JM, Ollerton J, Timmermann A, Andersen LH, Tossas AG (2009) Plant-hummingbird interactions in the West Indies: floral specialisation gradients associated with environment and hummingbird size. Oecologia 159: 757-766.

15. Brown JH, Kodric-Brown A (1979) Convergence, competition, and mimicry in a temperate community of hummingbird-pollinated flowers. Ecology 60: 1022-1035.

16. Momose K, Yumoto T, Nagamitsu T, Kato M, Nagamasu H, et al. (1998) Pollination biology in a lowland dipterocarp forest in Sarawak, Malaysia. 1. Characteristics of the plant-pollinator community in a lowland dipterocarp forest. Am J Bot 85: 1477-1501.

17. Piacentini VdQ, Varassin IG (2007) Interaction network and the relationships between bromeliads and hummingbirds in an area of secondary Atlantic rain forest in southern Brazil. J Trop Ecol 23: 663-671.

18. Ramirez N (1989) Biologia de polinización en una comunidad arbustiva tropical de la Alta Guayana Venezolana. Biotropica 21: 319-330.

19. Stiles FG (1975) Ecology, flowering phenology, and hummingbird pollination of some Costa Rican *Heliconia* species. Ecology 56: 285-301.

20. Vázquez DP, Simberloff D (2003) Changes in interaction biodiversity induced by an introduced ungulate. Ecol Lett 6: 1077-1083.

21. Wolf LL, Stiles FG, Hainsworth FR (1976) Ecological organization of a tropical, highland hummingbird community. J Anim Ecol 45: 349-379.

22. Ramirez N, Brito Y (1992) Pollination biology in a palm swamp community in the Venezuelan central plains. Bot J Linn Soc 110: 277-302.

23. Whelan RJ, Burbidge AH (1980) Flowering phenology, seed set and bird pollination of five Western Australian *Banksia* species. Aust J Ecol 5: 1-7.

24. Geerts S, Pauw A (2009) Hyper-specialization for long-billed bird pollination in a guild of South African plants: the Malachite Sunbird pollination syndrome. S Afr J Bot 75: 699-706.

25. Fleming TH, Tuttle MD, Horner MA (1996) Pollination biology and the relative importance of nocturnal and diurnal pollinators in three species of Sonoran Desert columnar cacti. Southwest Nat 41: 257-269.

26. DeWitt Smith S, Ané C, Baum DA (2008) The role of pollinator shifts in the floral diversification of *Iochroma* (Solanaceae). Evolution 62: 793-806.

27. Wolfe LM, Sowell DR (2006) Do pollination syndromes partition the pollinator community? A test using four sympatric morning glory species. Int J Plant Sci 167: 1169-1175.

28. Franceschinelli EV (2005) The pollination biology of two species of *Helicteres* (Malvaceae) with different mechanisms of pollen deposition. Flora 200: 65-73.

29. Slauson LA (2000) Pollination biology of two chiropterophilous agaves in Arizona. Am J Bot 87: 825-836.

30. de Waal C (2010) Reproductive ecology of bird-pollinated *Babiana* (Iridaceae): floral variation, mating patterns and genetic diversity. MS Thesis. University of Toronto. 158 pp.

31. Weston KA, Chapman HM, Kelly D, Moltchanova EV (2012) Dependence on sunbird pollination for fruit set in three West African montane mistletoe species. J Trop Ecol 28: 205-213.

32. Goldblatt P, Manning JC (2007) Floral biology of *Babiana* (Iridaceae: Crocoideae): Adaptive floral radiation and pollination. Ann Miss Bot Gard 94: 709-733.

33. Valiente-Banuet A, Rojas-Martínez A, Casas A, del Coro Arizmendi M, Dávila P (1997) Pollination biology of two winter-blooming giant columnar cacti in the Tehuacán Valley, central Mexico. J Arid Environ 37: 331-341.

34. Nassar JM, Ramírez N, Linares O (1997) Comparative pollination biology of Venezuelan columnar cacti and the role of nectar-feeding bats in their sexual reproduction. Am J Bot 84: 918-927.

35. Ortiz­Pulido R, Díaz SA, Valle-Díaz OI, Fisher AD (2012) Hummingbirds and the plants they visit in the Tehuacán-Cuicatlán Biosphere Reserve, Mexico. Rev Mex Biodivers 83: 152-163.

36. Freitas L, Sazima M (2006) Pollination biology in a tropical high-altitude grassland in Brazil: interactions at the community level. Ann Miss Bot Gard 93: 465-516.

37. Medan D, Montaldo NH, Devoto M, Mantese A, Vasellati V, Roitman GG, Bartoloni NH (2002) Plant-pollinator relationships at two altitudes in the Andes of Mendoza, Argentina. Arctic Antarctic Alpine Res 34: 233-241.

38. Carlo TA, Collazo JA, Groom MJ (2003) Avian fruit preferences across a Puerto Rican forested landscape: pattern consistency and implications for seed removal. Oecologia 134: 119-131.

39. Hammann A, Curio B (1999) Interactions among frugivores and fleshy fruit trees in a Philippine submontane rainforest. Conserv Biol 13: 766-773.

40. Mack AL, Wright DD (1996) Notes on occurrence and feeding of birds at Crater Mountain Biological Research Station, Papua New Guinea. Emu 96: 89-101.

41. Noma N (1997) Annual fluctuations of sapfruits production and synchronization within and inter species in a warm temperate forest on Yakushima Island, Japan. Tropics 6: 441-449.

42. Snow BK, Snow DW (1988) Birds and berries: a study of an ecological interaction. London: Poyser. 268 p.

43. Snow BK, Snow DW (1971) The feeding ecology of tanagers and honeycreepers in Trinidad. Auk 88: 291-322.

44. Sorensen AE (1981) Interactions between birds and fruit in a temperate woodland. Oecologia 50: 242-249.

45. Bleher B, Böhning-Gaese K (2001) Consequences of frugivore diversity for seed dispersal, seedling establishment and the spatial pattern of seedlings and trees. Oecologia 129: 385-394.

46. Baird JW (1980) The selection and use of fruit by birds in an eastern forest. Wilson Bull 92: 63-73.

47. Frost PGH (1980) Fruit-frugivore interactions in a South African coastal dune forest. In Noring R, editor. Acta XVII Congressus Internationalis Ornithologici. Berlin, Germany: Deutsche Ornithologische Ges. pp. 1179-1184.

48. Guitián J (1983) Relaciones entre los frutos y los passeriformes en un bosque montano de la Cordillera Cantábrica Occidental. PhD Thesis. Santiago, Spain: Universidad de Santiago.

49. Galetti M, Pizo MA (1996) Fruit eating birds in a forest fragment in southeastern Brazil. Ararajuba, Rev Bras Ornitol 4: 71-79.

50. Rezende EL, Lavabre JE, Guimarães P, Jordano P, Bascompte J (2007) Non-random coextinctions in phylogenetically structured mutualistic networks. Nature 448: 925-928.

51. Bascompte J, Jordano P, Oleson JM (2006) Asymmetric coevolutionary networks facilitate biodiversity maintenance. Science 312: 431-433.

52. Herrera CM (1984) A study of avian frugivores, bird-dispersed plants, and their interaction in Mediterranean scrublands. Ecol Monogr 54: 1-23.

53. Kantak GE (1979) Observations on some fruit-eating birds in Mexico. Auk 96: 183-186.

54. Lambert F (1989) Fig-eating by birds in a Malaysian lowland rain forest. J Trop Ecol 5: 401-412.

55. Sanitjan S, Chen J (2009) Habitat and fig characteristics influence the bird assemblage and network properties of fig trees from Xishuangbanna, South-West China. J Trop Ecol 25: 161-170.

56. Schleuning M, Blüthgen N, Flörchinger JB, Schaefer HM, Böhning-Gaese K (2011) Specialization and interaction strength in a tropical plant-frugivore network differ among forest strata. Ecology 92: 26-36.

57. Silva WR, Marco PD, Hasui E, Gomes VSM (2002) Patterns of fruit-frugivores interactions in two Atlantic Forest bird communities of south-eastern Brazil: implications for conservation. In Levey DJ, Silva WR, Galetti M, editors. Seed dispersal and frugivory: ecology, evolution and conservation. Wallingford: CAB International. pp 423-435.

58. Wheelwright NT, Haber WA, Murray KG, Guindon C (1984) Tropical fruit-eating birds and their food plants: a survey of a Costa Rican lower montane forest. Biotropica 16: 173-192.

59. Poulin B, Wright SJ, Lefebvre G, Calderon O (1999) Interspecific synchrony and asynchrony in the fruiting phenologies of congeneric bird-dispersed plants in Panama. J Trop Ecol 15: 213-227.

60. Engel TR (2000) Seed dispersal and forest regeneration in a tropical lowland biocoenosis (Shimba Hills, Kenya). PhD dissertation. Bayreuth, Germany: University of Bayreuth.

61. Stiles EW (1979) Notes on the natural history of *Heliconia* (Musaceae) in Costa Rica. Brenesia 15: 151-180.

62. Debussche M, Isenmann P (1989) Fleshy fruit characters and the choices of bird and mammal seed dispersers in a Mediterranean region. Oikos 56: 327-338.

63. Malmborg PK, Willson MF (1988) Foraging ecology of avian frugivores and some consequences for seed dispersal in an Illinois woodlot. Condor 90: 173-186.

64. Izhaki I, Walton PB, Safriel UN (1991) Seed shadows generated by frugivorous birds in an eastern Mediterranean scrub. J Ecol 79: 575-590.

65. Sahley CT (1996) Bat and hummingbird pollination of an autotetraploid columnar cactus, *Weberbauerocereus weberbaueri* (Cactaceae). Am J Bot 83: 1329-1336.

66. Waser NM (1978) Competition for hummingbird pollination and sequential flowering in two Colorado wildflowers. Ecology 59: 934-944.

67. Menges E (1995) Factors limiting fecundity and germination in small populations of *Silene regia* (Caryophyllaceae), a rare hummingbird-pollinated prairie forb. Am Midl Nat 133: 242-255.

68. Navarro L (1999) Pollination ecology and effect of nectar removal in *Macleania bullata* (Ericaceae). Biotropica 31: 618-625.

69. Lange RS, Scobell SA, Scott PE (2000) Hummingbird-syndrome traits, breeding system, and pollinator effectiveness in two syntopic penstemon species. Int J Plant Sci 161: 253-263.

70. Reid WH, Sensiba P, Freeman CE (1988) A mixed pollination system in *Penstemon pseudospectabilis* M. E. Jones (Scrophulariaceae). West N Am Naturalist 48: 489-494.

71. Sahley CT (2001) Vertebrate pollination, fruit production, and pollen dispersal of *Stenocereus thurberi* (Cactaceae). Southwest Nat 46: 261-271.

72. Dar S, del Coro Arizmendi M, Valiente-Banuet A (2006) Diurnal and nocturnal pollination of *Marginatocereus marginatus* (Pachycereeae: Cactaceae) in central Mexico. Ann Bot-London 97: 423-427.

73. Schmid S, Schmid VS, Zillikens A, Harter-Marques B, Steiner J (2011) Bimodal pollination system of the bromeliad *Aechmea nudicaulis* involving hummingbirds and bees. Plant Biol 13 (Suppl. 1): 41-50.

74. Cunningham SA (1991) Experimental evidence for pollination of *Banksia* spp. by non-flying mammals. Oecologia 87: 86-90.

75. Ramsey MW (1988) Difference in pollinator effectivenes of birds and insects visiting *Banksi menziesii* (Proteaceae). Oecologia 76: 119-124.

76. Vaughton G (1996) Pollination disruption by European honeybees in the Australian bird-pollinated shrub *Grevillea barklyana* (Proteaceae). Plant Syst Evol 200, 89-100.

77. Crome FHJ, Irvine AK (1986) “Two bob each way”: the pollination and breeding system of the Australian ran forest tree *Syzygium cormiflorum* (Myraceae). Biotropica 18: 115-125.

78. Botes C, Johnson SD, Cowling RM (2009) The birds and the bees: using selective exclusion to identify effective pollinators of African tree aloes. Int J Plant Sci 170: 151-156.

79. Schmidt-Adam G, Murray BG, Young AG (2009) The relative importance of birds and bees in the pollination of *Metrosideros excelsa*. Austral Ecol 34: 490-498.

80. Hargreaves AL, Johnson SD, Nol E (2004) Do floral syndromes predict specialization in plant pollination systems? An experimental test in an “ornithophilous” African *Protea*. Oecologia 140: 295-301.

81. Kalinganire A, Harwood CE, Slee MU, Simons AJ (2001) Pollination and fruit-set of *Grevillea robusta* in western Kenya. Austral Ecol 26: 637-648.

82. Vanstone VA, Paton DC (1988) Extrafloral nectaries and pollination of *Acacia pycnantha* Benth. by birds. Aust J Bot 36: 519-531.

83. Kunitake YK, Hasegawa M, Miyashita T, Higuchi H (2004) Role of a seasonally specialist bird *Zosterops japonica* on pollen transfer and reproductive success of *Camellia japonica* in a temperate area. Plant Spec Biol 19: 197-201.

84. Rodríguez-Rodríguez MC, Valido A (2008) Opportunistic nectar-feeding birds are effective pollinators of bird-flowers from Canary Islands: experimental evidence from *Isoplexis canariensis* (Scrophulariaceae). Am J Bot 95: 1408-1415.

85. Itino T, Kato M, Hotta M (1991) Pollination ecology of the two wild bananas, *Musa acuminata* subsp. *halabanensis* and *M. salaccensis*: chiropterophily and ornithophily. Biotropica 23: 151-158.

86. Geerts S, Pauw A (2009) African sunbirds hover to pollinate an invasive hummingbird-pollinated plant. Oikos 118: 573-579.

87. Kleizen C, Midgley J, Johnson SD (2008) Pollination systems of *Colchicum* (Colchicaceae) in southern Arica: evidence for rodent pollination. Ann Bot-London 102: 747-755.

88. Wiens D, Rourke JP, Casper BB, Rickart EA, LaPine TR, et al. (1983) Nonflying mammal pollination of southern African proteas: a non-coevolved system. Ann Mo Bot Gard 70: 1-31.

89. Johnson SD, Pauw A, Midgley J (2001) Rodent pollination in the African lily *Massonia depressa* (Hyacinthaceae). Am J Bot 88: 1768-1773.

90. Letten AD, Midgley JJ (2009) Rodent pollination in the Cape legume *Liparia parva*. Austral Ecol 34: 233-236.

91. Hackett DJ, Goldingay RL (2001) Pollination of *Banksia* spp. by non-flying mammals in north-eastern New South Wales. Aust J Bot 49: 637-644.

92. Goldingay RL, Carthew SM, Whelan RJ (1991) The importance of non-flying mammals in pollination. Oikos 61: 79-87.

93. Carthew SM (1993) An assessment of pollinator visitation to *Banksia spinulosa*. Aust J Ecol 18: 257-268.

94. Wooller RD, Wooller SJ (2003) The role of non-flying animals in the pollination of *Banksia nutans*. Aust J Bot 51: 503-507.

95. Nathan PT, Raghuram H, Elangovan V, Karuppudurai T, Marimuthu G (2005) Bat pollination of kapok tree, *Ceiba pentandra*. Curr Sci India 88: 1679-1681.

96. Nathan PT, Karuppudurai T, Raghuram H, Marimuthu G (2009) Bat foraging strategies and pollination of *Madhuca latifolia* (Sapotaceae) in southern India. Acta Chiropterol 11: 435-441.

97. Bumrungsri S, Harbit A, Benzie C, Carmouche K, Sridith K, et al. (2001) The pollination ecology of chiropterophilous canopy trees, *Parkia speciosa* Hassk. and *P. timoriana* (DC.) Merr. (Mimosaceae) in southern Thailand. Pittani, Thailand: Prince of Songkla University.

98. Méndez M. Durán R, Dorantes A, Dzib, G, Simá L, et al. (2005) Floral demography and reproductive system of *Pterocereus gaumeri*, a rare columnar cactus endemic to Mexico. J Arid Environ 62: 363-376.

99. Petit S. (1995) The pollinators of two species of columnar cacti on Curacao, Netherlands Antilles. Biotropica 27: 538-541.

100. Rocha M, Valera A, Eguiarte LE (2005) Reproductive ecology of five sympatric *Agave Littaea* (Agavaceae) species in central Mexico. Am J Bot 92: 1330-1341.

101. Cunningham SA (1996) Pollen supply limits fruit initiation by a rain forest understorey palm. J Ecol 84: 185-194.

102. Casas A, Valiente-Banuet A, Rojas-Martínez A, Dávila P (1999) Reproductive biology and the process of domestication of the columnar cactus *Stenocereus stellatus* in central Mexico. Am J Bot 86: 534-542.

103. Valiente-Banuet A, Molina-Freaner F, Torres A, Arizmendi MdC, Casas A (2004) Geographic differentiation in the pollination system of the columnar cactus *Pachycereus* *pecten-aboriginum*. Am J Bot 91: 850-855.

104. Ibarra-Cerdeña CN, Iñiguez-Dávalos LI, Sánchez-Cordero V (2005) Pollination ecology of *Stenocereus queretaroensis* (Cactaceae), a chiropterophilous columnar cactus, in a tropical dry forest of Mexico. Am J Bot 92: 503-509.

105. Nassar JM, Ramírez N, Linares O (1997) Comparative pollination biology of Venezuelan columnar cacti and the role of nectar-feeding bats in their sexual reproduction. Am J Bot 84: 918-927.

106. Molina-Freaner F, Rojas-Martínez A, Fleming TH, Valiente-Banuet A (2004) Pollination biology of the columnar cactus *Pachycereus pecten-aboriginum* in north-western Mexico. J Arid Environ 56: 117-127.

107. Fleming TH, Sahley CT, Holland JN, Nason JD, Hamrick JL (2001) Sonoran Desert columnar cacti and the evolution of generalized pollination systems. Ecol Monogr 71: 511-530.

108. Hansen DM, Müller CB (2009) Invasive ants disrupt gecko pollination and seed dispersal of the endangered plant *Roussea simplex* in Mauritius. Biotropica 41: 202-208.

109. Traveset A, Sáez E. (1997) Pollination of *Euphorbia dendroides* by lizards and insects: spatio-temporal variation in patterns of flower visitation. Oecologia 111: 241-248.

110. Pérez-Mellado V, Casas JL (1997) Pollination by a lizard on a Mediterranean island. Copeia 1997: 593-595.
